# Comparison of KONAR-multifunctional occluder and Amplatzer occluders in transcatheter closure of patent arterial ducts and ventricular septal defects

**DOI:** 10.1186/s43044-026-00768-x

**Published:** 2026-07-22

**Authors:** Fatma Aboalsoud Taha, Marwa Desoky Abohamar, Raghda Ghonimy Elsheikh, Nouran Mostafa Mansour

**Affiliations:** Cardiology Department, Tanta University, Tanta, Egypt

**Keywords:** Patent ductus arteriosus, Ventricular septal defect, Transcatheter closure, KONAR-multifunctional occluder, Amplatzer devices

## Abstract

**Background:**

Transcatheter closure of patent ductus arteriosus (PDA) and ventricular septal defect (VSD) is performed using various occluder devices.

**Objective:**

We aim to compare the use of KONAR-Multifunctional Occluder (KONAR-MFO) and Amplatzer occluders (ADO-I, ADO-II) for closure of PDAs and VSDs.

**Methods:**

This is a retrospective study on patients who underwent transcatheter PDA or VSD closure between June 2023 and October 2025. Patients were categorized according to the occlusion device and the underlying defect. Data were analysed comprehensively.

**Results:**

The study included 173 patients; 116 (67.1%) and 57 (32.9%) underwent PDA and VSD closure, respectively, using 99 ADO-I, 53 KONAR-MFO, and 21 ADO-II devices. The KONAR-MFO was used in smaller, younger patients, and the ADO-I in heavier, older patients (*p* < 0.05). The ADO-I was used for PDA type-A (40.4%), type-E (12.1%), type-C and D (9.1% for each), as well as perimembranous and muscular VSDs (13.1% each). The KONAR-MFO was used for peri-membranous VSDs (47.2%), Gerbode-type VSDs (5.7%), and PDA type-B (14.1%). The ADO-II was employed for PDA type-E (57.1%) and type-C (42.9%), without use in VSDs. The KONAR-MFO and ADO-II devices showed the shortest procedural and fluoroscopy times with the lowest contrast use, owing to single-access deployment without arteriovenous loop formation. Freedom from all types of complications was 73.9%, 71.1%, and 66.7% for KONAR-MFO, ADO-I, and ADO-II, respectively. In ADO-I, residual shunt, device embolization, and complete heart block occurred in 2.0% each. In KONAR-MFO, left pulmonary artery (LPA) stenosis and mild aortic regurgitation were observed in 3.8% each, while residual shunt and device embolization were observed in 1.9%. ADO-II was associated with the highest residual shunt and device embolization, both occurring at 9.5%.

**Conclusions:**

ADO-I was used to close PDA and VSD, with longer procedural times. KONAR-MFO offered useful applications across complex VSDs with safety profiles in smaller, younger patients. ADO-II was preferred for tubular and elongated PDAs.

## Introduction

Congenital heart defects (CHDs) such as patent ductus arteriosus (PDA) and ventricular septal defect (VSD) are among the most common structural cardiac anomalies requiring intervention in both pediatric and adult populations. Traditionally managed via open-heart surgery, the evolution of transcatheter closure techniques has transformed management strategies, offering less invasive options with reduced morbidity and hospital stay [1]. 

The Amplatzer family of occluder devices (Amplatzer Duct Occluder series) (Abbott Vascular, USA) has long been considered the gold standard for transcatheter closure of PDAs and VSDs due to their proven efficacy and high success rates. These devices are characterized by their nitinol design and polyester fabric, promoting rapid endothelization and effective occlusion [2, 3]. 

In recent years, the KONAR-multifunctional occluder (KONAR-MFO) (LifeTech Scientific, China) has emerged as a promising alternative, especially in settings requiring flexible deployment and low-profile devices. Developed with a softer nitinol mesh and a double screw hybrid delivery system (allowing both antegrade and retrograde approaches), the KONAR-MFO has shown comparable closure rates with shorter procedural and fluoroscopy times in certain studies [4, 5]. Importantly, its softer configuration and reduced radial force may offer a lower risk of conduction disturbances, a key concern in perimembranous VSD closure [6–8]. 

Despite individual device reports, to our knowledge, comparisons between KONAR-MFO and Amplatzer-type devices remain limited in the literature, particularly across different age groups and lesion types. Therefore, this study aims to compare the efficacy, safety, and procedural characteristics of KONAR-MFO versus Amplatzer occluders in the closure of PDAs and VSDs in both children and adults.

## Methods

### Study design and population

This is a retrospective observational study that enrolled all patients who had either a PDA or a VSD and were deemed suitable for transcatheter closure between June 2023 and October 2025 using either the Amplatzer device family or the KONAR-MFO.

Patients were eligible for inclusion with the following criteria: (1) Symptomatic presentation (dyspnea, fatigue, arrhythmia, or chest pain). (2) Evidence of hemodynamically significant PDA or VSD. (3) Anatomical suitability for device closure as confirmed by transthoracic echocardiography and/or angiography. (4) Appropriate for closure using either Amplatzer devices [Amplatzer duct occluder I (ADO-I), Amplatzer duct occluder II (ADO-II)] or KONAR-MFO. Initially, patients were categorized according to the occlusion device. Subsequently, to minimize lesion-related heterogeneity and enable meaningful comparisons, the study population was stratified into two subgroups based on the underlying cardiac defect. Comprehensive subgroup analyses, including baseline characteristics, anatomical features, procedural parameters, outcomes, and device-related complications, were conducted.

Patients with active endocarditis, intracardiac thrombus, complex CHDs requiring surgical repair, severe pulmonary hypertension, and inadequate vascular access were excluded from the study.

The study was conducted in accordance with the ethical standards of our institutional research committee, with a registration protocol ID of 36264PR1356/8/25, and with the 1964 Helsinki ethical declaration and its later amendments. Informed written consent was obtained from all participants or their legal guardians in the study for the procedure, for participation in the study, and for publishing their data. Any unexpected risks were cleared with them, and the ethical committee ensured the privacy and confidentiality of the participants’ data.

### Procedure and device selection

Under general anesthesia, prophylactic antibiotics and heparin (50–100 IU/Kg) were administered. All procedures were performed under fluoroscopic and echocardiographic guidance, using standard catheterization techniques. The size and the anatomy of the defect were assessed by angiography/ventriculography. Device selection was based on the anatomical characteristics of the defect, adherence to the adjacent structures, and the operator’s discretion. Selecting the correct size for the PDA involves measuring the narrowest diameter of the PDA (often at the pulmonary end), and for the VSD involves the narrowest part of the VSD (usually the RV exit), via angiography and echocardiography. All measurements were performed in diastole. As a general rule, the device is chosen to be larger than the narrowest diameter, with specific criteria for different device types; for ADO-I and KONAR-MFO: 1–2 mm larger than the narrowest diameter of the duct or the VSD, for ADO-II device: 2–3 mm larger than the narrowest diameter of the duct. ADO-II is often used for small ducts < 3.5 mm. Device placement was confirmed by control angiography/ventriculography and post-deployment echocardiography. All patients were monitored post-procedure for complications. Two to three mg/kg/day of acetylsalicylic acid (ASA) was prescribed for all patients and continued for 6 months post-procedure.

### Outcome measures

The primary outcome was procedural success, defined as the successful device deployment in the intended position without the need for surgical intervention and with immediate adequate defect closure. Secondary outcomes included: presence of residual shunt, device embolization, transient or persistent arrhythmias, valvular interference, or the need for reintervention.

### Follow-up

All patients were clinically evaluated and underwent transthoracic echocardiography at 24 h post-procedure to assess device position, residual shunt, and cardiac function.

### Statistical analysis

Data were collected and analyzed using SPSS Statistics Software version 28.0. Continuous variables were presented as mean ± standard deviation (SD), as appropriate. Categorical variables were expressed as frequencies and percentages. Comparisons between (Amplatzer devices vs. KONAR-MFO) were performed using the Kruskal–Wallis Test (K) for continuous data, and the chi-square for categorical data. Post hoc (Scheffé) test was used when a statistically significant overall difference had been identified among the device cohorts. A p-value < 0.05 was considered statistically significant.

## Results

### Patient demographics and baseline characteristics

A total of 173 patients were included in the study. 116 (67.1%) patients underwent transcatheter PDA closure, and 57 (32.9%) patients underwent transcatheter VSD closure using 99 ADO-I, 53 KONAR-MFO, and 21 ADO-II devices. Baseline characteristics, including age, gender, and weight, are presented in Table 1.

Patients with the ADO-I device were the oldest (27.4 ± 12.4 years), followed by those with the ADO-II device (5.7 ± 5.9 years), while patients with the KONAR-MFO device were the youngest (1.9 ± 0.9 years) (*p* < 0.05). Similarly, body weight differed significantly among the cohort, mirroring the age distribution (*p* < 0.001). Gender distribution was not varied (*P* = 0.132), with most patients being females.

### Clinical presentation and hemodynamic parameters

As shown in Table 1, there were statistically significant differences in clinical symptoms among the three device cohorts (*p* < 0.001).

The ADO-I patients had the highest proportion of symptoms, with common presentations including recurrent chest infections (37.4%), shortness of breath (25.3%), and failure to thrive (11.1%). In contrast, the KONAR-MFO and ADO-II patients demonstrated significantly greater proportions of asymptomatic patients, at 45.3% and 66.7%, respectively (*p* < 0.001). Notably, palpitations were reported in 3% of ADO-I patients, but were absent with the other devices.

Regarding the pulmonary-to-systemic flow (Qp/Qs) ratio, the ADO-I patients had the highest mean Qp/Qs ratio (2.2 ± 0.4), followed by the KONAR-MFO patients (2.0 ± 0.3), and the ADO-II patients (1.7 ± 0.3), *p* = 0.004. Post hoc comparisons indicated a significant difference between ADO-I and ADO-II (*p* = 0.004), while comparisons between ADO-I and KONAR-MFO (*p* = 0.328) and between KONAR-MFO and ADO-II (*p* = 0.095) were not statistically significant.

The mean systolic pulmonary artery pressure (SPAP) values were comparable across the ADO-I (37.1 ± 8.0 mmHg), KONAR-MFO (38.8 ± 6.2 mmHg), and ADO-II (34.0 ± 6.2 mmHg) cohorts, with no statistically significant difference (*p* = 0.081).

**Table 1:** Comparison between the three devices regarding demographic and diagnostic data

Variables	ADO-I (*n* = 99)	KONAR-MFO (*n* = 53)	ADO-II (*n* = 21)	*P*-value
Age (years)	27.4 ± 12.45	1.9 ± 0.9	5.7 ± 5.9	< 0.001*
Post hoc (Scheffe test)	P1	P2	P3	
	< 0.001*	< 0.001*	0.002*	
Weight (Kg)	43.9 ± 10.7	14.3 ± 5.4	23.9 ± 10.7	< 0.001*
Post hoc (Scheffe test)	P1	P2	P3	
	< 0.001*	< 0.001*	0.226	
Female gender	76 (76.8%)	44 (83.0%)	16 (76.2)	0.132
Symptoms [n (%)]				< 0.001*
Asymptomatic	23 (23.2%)	24 (45.3%)	14 (66.7%)	
Failure to thrive	11 (11.1%)	6 (11.3%)	1 (4.8%)	
SOB	25 (25.3%)	7 (13.2%)	4 (19.0%)	
Recurrent chest infection	37 (37.4%)	16 (30.2%)	2 (9.5%)	
Palpitation	3 (3.0%)	0 (0.0%)	0 (0%)	
Qp/Qs ratio	2.2 ± 0.4	2.0 ± 0.3	1.7 ± 0.3	0.004*
Post hoc (Scheffe test)	P1	P2	P3	
	0.328	0.095	0.004*	
SPAP (mmHg)	37.1 ± 8.0	38.8 ± 6.2	34.0 ± 6.2	0.081

*ADO-I* Amplatzer duct occluder I, *ADO-II* Amplatzer duct occluder II, *KONAR-MFO* KONAR-Multifunctional Occluder, *Qp/Qs ratio* Pulmonary-to-systemic flow ratio, *SOB* Shortness of breath, *SPAP* Systolic pulmonary artery pressure, *K* Kruskal Wallis test *χ*^*2*^ Chi square test *P* Significant at ≤ 0.05, *P1* ADO-Ⅰ patients versus KONAR-MFO patients, *P2* KONAR-MFO patients versus ADO-Ⅱ patients, *P3* ADO-Ⅰ patients versus ADO-Ⅱ patients

### Procedural access routes

As detailed in Table 2, there was a highly significant difference in the type of vascular access used across the three device cohorts (*p* < 0.001).

The ADO-I patients were almost exclusively treated with combined arterial and venous access (83.8%), reflecting its traditional deployment requirements, whereas venous-only access was used in the remaining patients (16.2%). In contrast, the KONAR-MFO and the ADO-II patients had a much more varied distribution of access routes. Notably, venous-only access was used in nearly half of KONAR-MFO patients (49.1%) and one-third of ADO-II patients (33.3%); meanwhile, arterial-only access was employed in one-third of KONAR-MFO patients (32.1%) and in nearly half of ADO-II patients (47.6%), indicating a trend toward using a minimally invasive venous-only approach with the KONAR-MFO.

### Anatomical defect types and device selection

A statistically significant difference in the distribution of congenital cardiac defect types was observed among the three device populations (*p* < 0.001), as shown in Table 2.

The ADO-I device was used across a broad range of defects, with the majority being conical PDA type A (40.4%). It was also used in other types of PDAs [elongated PDA type E (12.1%), tubular PDA type C, and saccular PDA type D in 9.1% for each], along with VSDs [peri-membranous and muscular VSDs in 13.1% for each]. In contrast, the KONAR-MFO device was primarily used for peri-membranous VSDs (47.2%) and window PDA type B (14.1%), with additional use in tubular PDA type C (13.2%) and elongated PDA type E (5.7%). Notably, the KONAR-MFO device was also used for Gerbode-type VSDs (5.7%), which were not addressed with the other devices. The ADO-II device was almost exclusively employed for PDA closures, particularly elongated PDA type E (57.1%) and tubular PDA type C (42.9%), with no use in VSD patients. This indicates a strong device preference or limitation to certain PDA morphologies.

### Procedural efficiency parameters

Statistically significant differences in contrast amount, fluoroscopy time, and total procedural time (*p* < 0.001), as shown in Table 2, indicate clear variation in procedural demands across devices.

Fluoroscopy time was longest with ADO-I (8.7 ± 0.9 min), shorter with the KONAR (6.4 ± 0.9 min), and shortest with the ADO-II (5.6 ± 1.0 min). The total procedural time followed a similar trend, with ADO-I requiring the longest duration (45.4 ± 2.9 min), followed by the KONAR (37.9 ± 2.8 min), and finally the ADO-II with the shortest procedural time (24.1 ± 4.3 min). All pairwise comparisons were statistically significant (*p* < 0.001), indicating a meaningful difference in procedural efficiency across devices. The contrast amount was significantly higher with the ADO-I (49.4 ± 4.2 ml) compared to both the KONAR (31.1 ± 2.7 ml) and the ADO-II (29.9 ± 2.3 ml). Post hoc analysis confirmed significant differences between ADO-I and KONAR (*p* < 0.001) and between ADO-I and ADO-II (*p* < 0.001), while the difference between KONAR and ADO-II was not statistically significant (*p* = 0.447).

### Procedural complications and safety outcomes

As shown in Table 2, across all cohorts, the majority of patients experienced no complications. The KONAR-MFO patients had the lowest complication rate, with 73.9% of patients being free of complications, followed by the ADO-I (71.1%) and the ADO-II (66.7%) cohorts. The ADO-I had the highest rate of access site hematoma (8.1%), likely due to more frequent use of combined arterial and venous access. It also showed instances of mild aortic coarctation (5.1%), residual shunt, device embolization, and complete heart block (CHB) in 2.0% for each. The KONAR-MFO showed isolated cases of femoral artery spasm, left pulmonary artery (LPA) stenosis, and mild aortic regurgitation in 3.8% for each, along with similar rates of residual shunt and device embolization in 1.9% for each. The ADO-II was associated with the highest femoral artery spasm, residual shunt, and device embolization, occurring at 9.5% for each.

**Table 2:** Comparison between the three devices regarding the procedural details

	ADO-I (*n* = 99)	KONAR-MFO (*n* = 53)	ADO-II (*n* = 21)	*P*
Access/approach				< 0.001*
Arterial & venous	83 (83.8%)	10 (18.9%)	4 (19.0%)	
Venous only	16 (16.2%)	26 (49.1%)	7 (33.3%)	
Arterial only	0 (0.0%)	17 (32.1%)	10 (47.6%)	
Anatomical defect type				< 0.001*
PDA type A (conical)	40 (40.4%)	0 (0.0%)	0 (0.0%)	
PDA type B (window)	0 (0.0%)	14 (14.1%)	0 (0.0%)	
PDA type C (tubular)	9 (9.1%)	7 (13.2%)	9 (42.9%)	
PDA type D (complex)	9 (9.1%)	1 (1.9%)		
PDA type E (elongated)	12 (12.1%)	3 (5.7%)	12 (57.1%)	
Peri-membranous VSD	13 (13.1%)	25 (47.2%)	0 (0.0%)	
High muscular VSD	6 (6.1%)	0 (0.0%)	0 (0.0%)	
Mid muscular VSD	7 (7.1%)	0 (0.0%)	0 (0.0%)	
Gerbode VSD	0 (0.0%)	3 (5.7%)	0 (0.0%)	
Apical VSD	3 (3.0%)	0 (0.0%)	0 (0.0%)	
Fluoroscopy time/min	8.7 ± 0.9	6.4 ± 0.9	5.6 ± 1.0	< 0.001*
Post hoc (Scheffe test)	P1	P2	P3	
	< 0.001*	0.980	< 0.001*	
Procedural time/min	45.4 ± 2.9	37.9 ± 2.8	24.1 ± 4.3	< 0.001*
Post hoc (Scheffe test)	P1	P2	P3	
	< 0.001*	< 0.001*	< 0.001*	
Contrast amount/ml	49.4 ± 4.2	31.1 ± 2.7	29.9 ± 2.3	< 0.001*
Post hoc (Scheffe test)	P1	P2	P3	
	< 0.001*	0.447	< 0.001*	
Procedural complications				0.04*
None	71 (71.1%)	39 (73.9%)	14 (66.7%)	
Access site hematoma	8 (8.1%)	0 (0.0%)	0 (0.0%)	
Femoral artery spasm	6 (6.1%)	2 (3.8%)	2 (9.5%)	
Mild aortic coarctation	5 (5.1%)	2 (3.8%)	1 (4.8%)	
LPA stenosis	0 (0.0%)	2 (3.8%)	0 (0.0%)	
Residual shunt	2 (2.0%)	1 (1.9%)	2 (9.5%)	
Device embolization	2 (2.0%)	1 (1.9%)	2 (9.5%)	
Complete heart block	2 (2.0%)	0 (0.0%)	0 (0.0%)	
Mild tricuspid regurgitation	3 (3.1%)	3 (5.7%)	0 (0.0%)	
Mild aortic regurgitation	0 (0.0%)	2 (3.8%)	0 (0.0%)	
Need for re-intervention	0 (0.0%)	0 (0.0%)	0 (0.0%)	

*ADO-I* Amplatzer duct occluder I, *ADO-II* Amplatzer duct occluder II, *KONAR-MFO* KONAR-Multifunctional Occluder, *LPA* Left pulmonary artery, *PDA* Patent ductus arteriosus, *VSD* Ventricular septal defect, *χ* ^*2*^Chi-square test *K* Kruskal-Wallis test *P* Significant at ≤ 0.05, *P1* ADO-Ⅰ patients versus KONAR-MFO patients, *P2* KONAR-MFO patients versus ADO-Ⅱ patients, *P3* ADO-Ⅰ patients versus ADO-Ⅱ patients

### Device size distribution

The most commonly used sizes of the ADO-I were 8/6 mm (39.4%) and 10/8 mm (29.3%), with less frequent use of sizes 12/10 mm (18.2%) and 6/4 mm (11.1%), and minimal use of 14/12 mm (2.0%). The KONAR-MFO displayed a wide range of device sizes, with the 9/7 mm (28.3%) size most frequently used, followed by 8/6 mm (18.9%) and 7/5 mm (17.7%). The availability and use of larger sizes (up to 16/14 mm) reflect the device’s versatility, particularly for closing perimembranous and other larger VSDs, including Gerbode defects. In contrast, the ADO-II had a more uniform distribution, with 5/4 mm (33.3%) and 6/4 mm (33.3%) being the most commonly used sizes. Other sizes, such as 4/4 mm (19.0%) and 6/6 mm (14.3%), were also used.

### PDA subgroup of patients (*n* = 116)

**Fig. 1:**
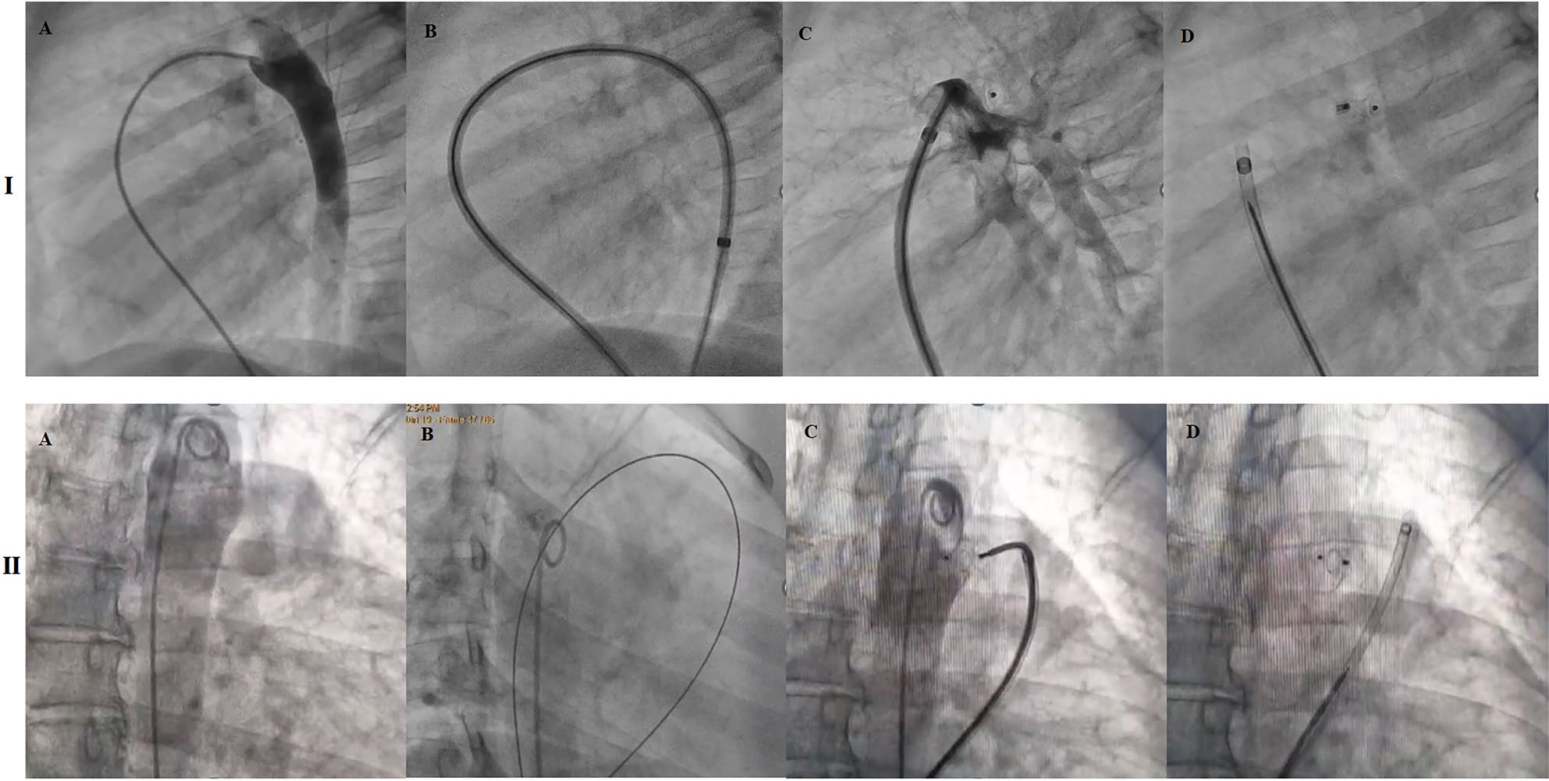
Transcatheter closure of a patent ductus arteriosus (PDA) with an Amplatzer duct occluder I (ADO-I). I: A 2.5-year-old female, 10 Kg, underwent transcatheter closure of a PDA through the venous-only approach. **A** Antegrade ascending thoracic aortogram with a 5 F MP catheter in lateral projection revealed a small conical Krichenko type A PDA with pulmonary end of 0.2 cm. **B** Antegrade crossing of a 5 F Amplatzer TorqVue 180 delivery through the PDA to the descending thoracic aorta over a 0.035/260 guidewire. **C** Antegrade deployment of a 6/4 mm ADO-I with pulmonary artery control through the delivery, denoting no residual shunt or encroachment on the left pulmonary artery or descending thoracic aorta. **D** Well-positioned device. II: A 26-year-old female underwent transcatheter closure of a PDA through the traditional approach. **A** Retrograde ascending thoracic aortogram with a 6 F PG catheter in right anterior oblique projection revealed a large conical Krichenko type A PDA with pulmonary end of 0.8 cm. **B** Antegrade crossing of a 7 F Amplatzer TorqVue 180 delivery through the PDA to the descending thoracic aorta over a 0.035-inch/260 stiff guidewire. **C** Antegrade deployment of a 12/10 mm ADO-I was under control through aortogram with a 6 F PG catheter, denoting no residual shunt or encroachment on the left pulmonary artery or descending thoracic aorta. **D** Well-positioned device

**Fig. 2:**
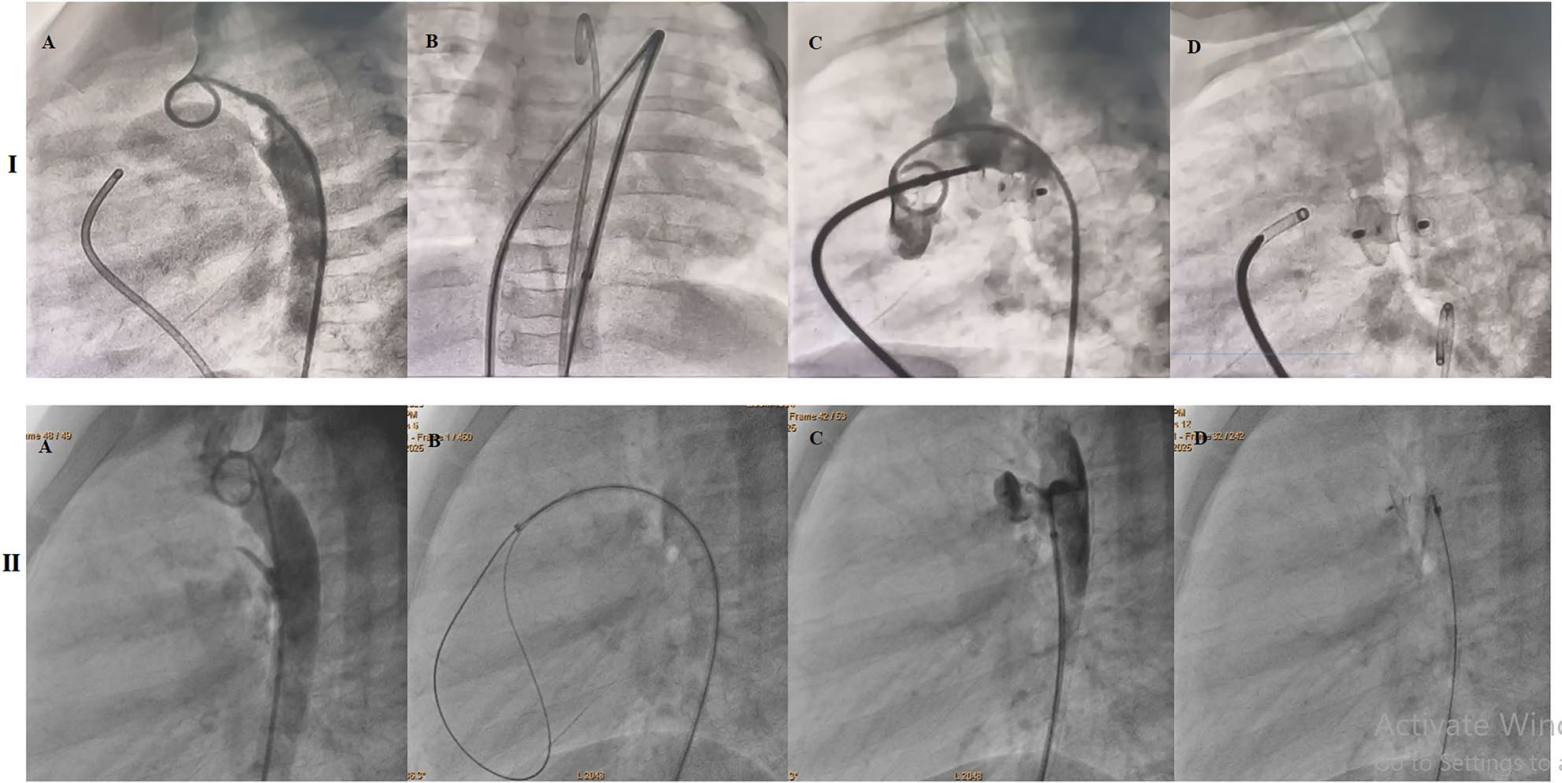
Transcatheter closure of a patent ductus arteriosus (PDA) with a KONAR-multifunctional occluder (KONAR-MFO). I: A 7-month-old female, 7 Kg, underwent transcatheter closure of a PDA through the traditional approach. **A** Retrograde ascending thoracic aortogram with a 5 F PG catheter in lateral projection revealed a large tubular, Krichenko type C PDA with a pulmonary end of 0.5 cm. **B** Antegrade crossing of a 6 F Lifetech delivery through the PDA to the descending thoracic aorta over a 0.035-inch/260 guidewire. **C** Antegrade deployment of a 9/7 mm KONAR-MFO with control descending thoracic aortogram through the 5 F PG catheter, denoting no residual shunt or encroachment on the left pulmonary artery or descending thoracic aorta. **D** Well-positioned device. II: A 3-year-old male underwent transcatheter closure of a PDA through the arterial approach. **A** Retrograde ascending thoracic aortogram with a 5 F PG catheter in lateral projection revealed a small, elongated Krichenko type E PDA with a pulmonary end of 0.2 cm. **B** Retrograde crossing of a 5 F MP catheter from the descending thoracic aorta to the pulmonary artery through the PDA over a 0.035/260 Terumo guidewire. **C** Retrograde crossing of a 5 F Lifetech delivery through the PDA over a 0.035-inch/260 guidewire. Then, retrograde deployment of a 6/4 mm KONAR-MFO with control descending thoracic aortogram through the delivery, denoting no residual shunt or encroachment on the left pulmonary artery or descending thoracic aorta. **D** Well-positioned device

**Fig. 3:**
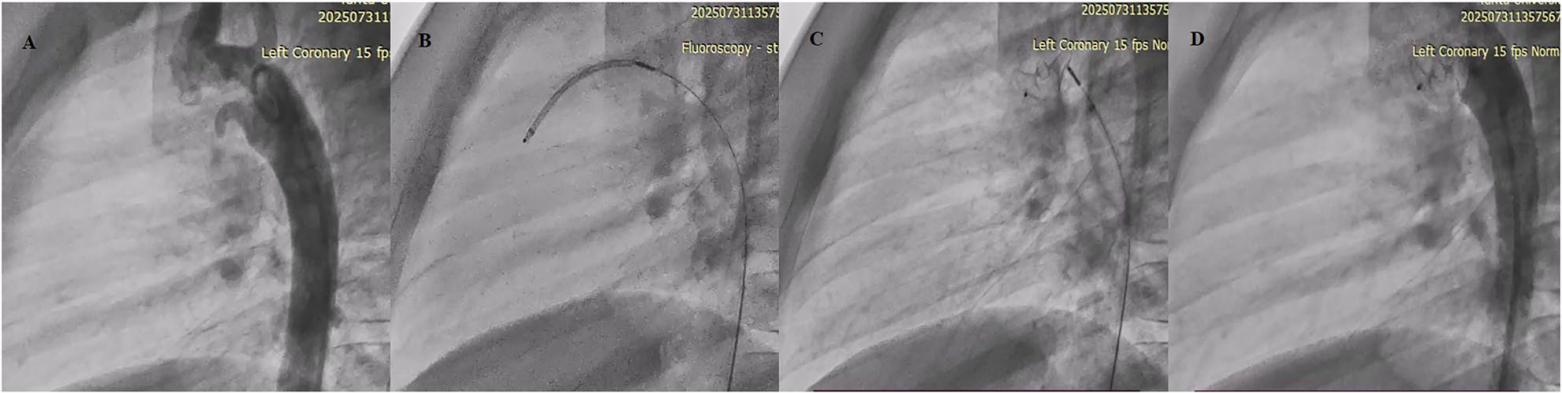
Transcatheter closure of a patent ductus arteriosus (PDA) with an Amplatzer duct occluder II (ADO-II). I: A 10-year-old female underwent transcatheter closure of a PDA through the arterial approach. **A** Retrograde ascending thoracic aortogram with a 5 F PG catheter in lateral projection revealed a small, elongated Krichenko type E PDA with a pulmonary end of 0.2 cm. **B** Retrograde crossing with a 5 F MP catheter from the descending thoracic aorta to the pulmonary artery through the PDA over a 0.035-inch/260 Terumo guidewire. Then, a retrograde crossing of a 5 F Lifetech delivery through the PDA over a 0.035-inch/260 guidewire. **C** Retrograde deployment of a 6/4 mm ADO-II. **D** Control descending thoracic aortogram through the delivery, denoting no residual shunt or encroachment on the left pulmonary artery or descending thoracic aorta. Well-positioned device

As shown in Table 3, PDA patients treated with ADO-I were significantly older and heavier than those receiving KONAR-MFO or ADO-II devices (*p* < 0.001). Device selection was strongly associated with PDA morphology (*p* < 0.001) (Figs. 1, 2 and 3). ADO-I was predominantly used for conical type A PDAs (57.1%), KONAR-MFO was most frequently utilized in window type B PDAs (56.0%), whereas ADO-II was mainly used for elongated type E (57.1%) and tubular type C PDAs (42.9%).

Regarding procedural performance, significant differences were observed in fluoroscopy time, total procedural time, and contrast volume (*p* < 0.001). ADO-I procedures required the greatest contrast volume and longest fluoroscopy and procedural times, whereas ADO-II demonstrated the shortest procedural metrics. KONAR-MFO showed intermediate procedural efficiency.

Regarding procedural safety outcomes, residual shunting and mild aortic coarctation occurred at similar rates, with no significant difference (*p* > 0.05). In contrast, device embolization was reported in 2 (9.5%) ADO-II patients, while LPA stenosis was reported in 2 (8.0%) KONAR-MFO patients, yielding a statistically significant difference (*p* < 0.05). Importantly, no patient required re-intervention, supporting the overall effectiveness and favorable safety profile of all devices despite the low incidence of device-related complications.

**Table 3:** Baseline and procedural characteristics of PDA patients (ADO-I vs. KONAR-MFO vs. ADO-II)

Variables	ADO-I (*n* = 70)	KONAR-MFO (*n* = 25)	ADO-II (*n* = 21)	*P*-value
Age (years)	23.7 ± 12.4	2.9 ± 0.7	4.3 ± 6.1	< 0.001*
Weight (kg)	39.4 ± 11.2	16.3 ± 6.1	22.5 ± 9.9	< 0.001*
Female gender	53 (75.7%)	20 (80.0%)	16 (76.2%)	0.232
Type A PDA	40 (57.1%)	0 (0.0%)	0 (0.0%)	< 0.001*
Type B PDA	0 (0.0%)	14 (56.0%)	0 (0.0%)	< 0.001*
Type C PDA	9 (12.9%)	7 (28.0%)	9 (42.9%)	< 0.001*
Type D PDA	9 (12.9%)	1 (4.0%)	0 (0.0%)	< 0.001*
Type E PDA	12 (17.1%)	3 (12.0%)	12 (57.1%)	< 0.001*
Fluoroscopy time (min)	7.9 ± 0.6	5.1 ± 0.8	5.2 ± 1.0	< 0.001*
Procedural time (min)	41.1 ± 2.5	36.2 ± 3.1	23.9 ± 3.4	< 0.001*
Contrast amount (ml)	48.3 ± 6.3	30.7 ± 1.7	29.3 ± 1.3	< 0.001*
Residual shunt	1 (1.4%)	0 (0.0%)	2 (9.5%)	0.345
Device embolization	0 (0%)	0 (0.0%)	2 (9.5%)	0.022*
Mild aortic coarctation	5 (7.1%)	2 (8.0%)	1 (4.8%)	0.411
LPA stenosis	0 (0.0%)	2 (8.0%)	0 (0.0%)	0.041*
Re-intervention	0 (0.0%)	0 (0.0%)	0 (0.0%)	

*ADO-I* Amplatzer duct occluder I, *ADO-II* Amplatzer duct occluder II, *KONAR-MFO* KONAR-Multifunctional Occluder, *LPA* Left pulmonary artery, *PDA* Patent ductus arteriosus, *Qp/Qs* Pulmonary-to-systemic flow ratio, *SPAP* Systolic pulmonary artery pressure,

P: Significant at ≤ 0.05

### VSD subgroup of patients (*n* = 57)

**Fig. 4:**
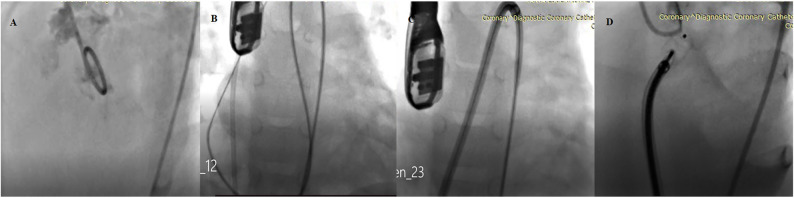
Transcatheter closure of a ventricular septal defect (VSD) with an Amplatzer duct occluder I (ADO-I). A 5-year-old female underwent transcatheter closure of a VSD through the traditional approach. **A** Retrograde left ventriculogram with a 5 F PG catheter in left anterior oblique projection revealed a perimembranous VSD with a right ventricular exit of 0.3 cm. **B** Retrograde crossing with a 5 F cut PG catheter through the VSD over a 0.035-inch/260 Terumo guidewire. Then, snaring of the Terumo guidewire in the superior vena cava was done to retrieve the wire from the venous access and induce an arteriovenous loop. **C** Antegrade crossing of a 6 F Amplatzer TorqVue 180 delivery through the VSD over a 0.035-inch/260 guidewire (Kissing delivery catheter technique). **D** Antegrade deployment of a 10/8 mm ADO-I device with control ascending aortogram with a 5 F PG catheter that revealed a well-positioned device, no residual shunt, and no encroachment on the aortic valve

**Fig. 5:**
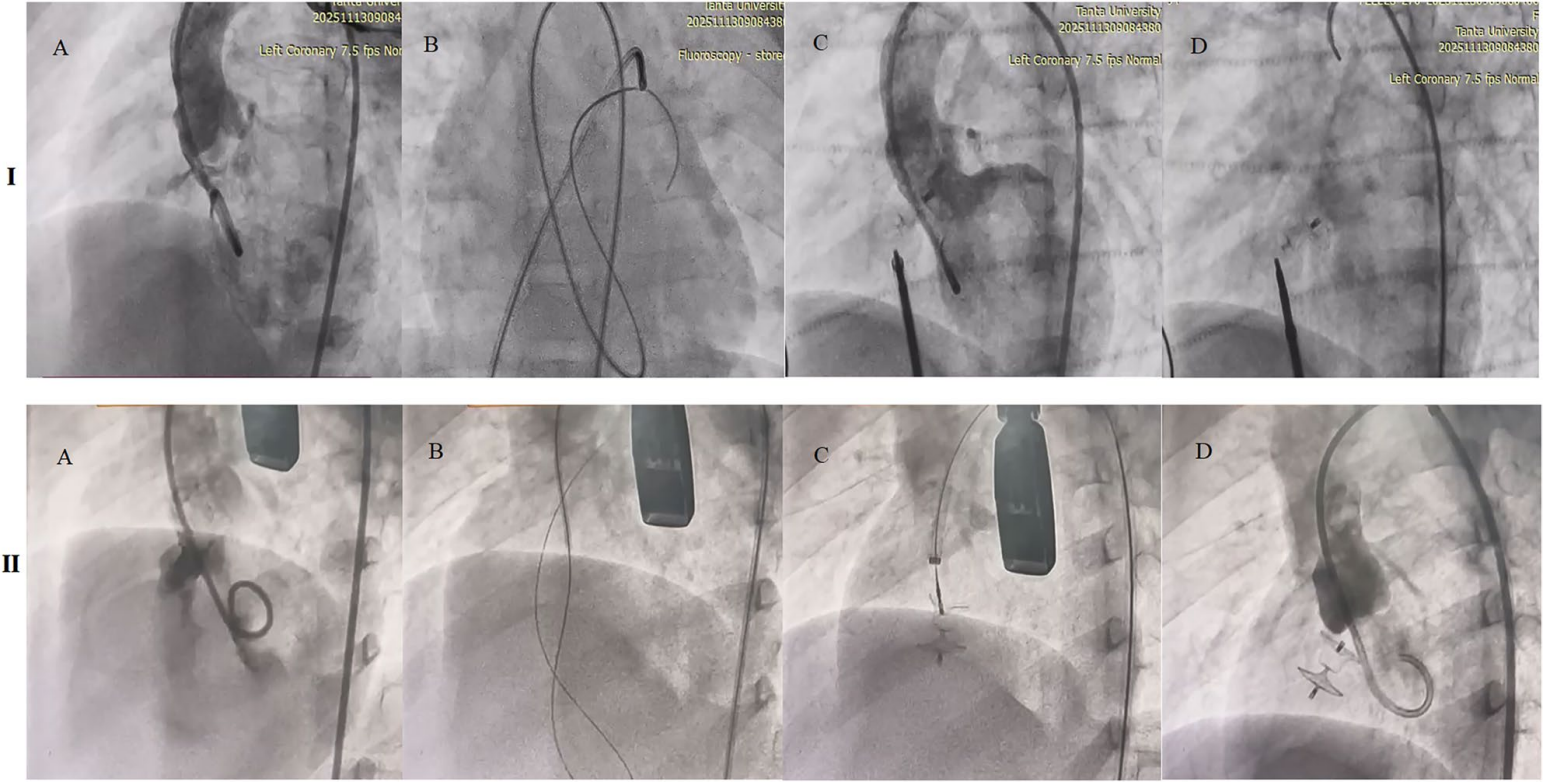
Transcatheter closure of a ventricular septal defect (VSD) with a KONAR-multifunctional occluder (KONAR-MFO). I: A 2-year-old female, 9 Kg, underwent transcatheter closure of a VSD through the traditional approach. **A** Retrograde left ventriculogram with a 5 F PG catheter in left anterior oblique projection revealed a perimembranous tunnel-shaped VSD with an inlet extension, a minute aortic rim, and a right ventricular exit of 0.3 cm. **B** Retrograde crossing with a 5 F cut PG catheter through the VSD over a 0.035-inch/260 Terumo guidewire. Then, snaring of the Terumo guidewire in the left pulmonary artery was done to retrieve the wire from the venous access and induce an arteriovenous loop. **C** Antegrade crossing of a 6 F Lifetech delivery through the VSD over a 0.035-inch/260 guidewire (Kissing delivery catheter technique). Then, an antegrade deployment of a 7/5 mm KONAR-MFO device. **D** Control ascending aortogram with a 5 F cut PG catheter that revealed a well-positioned device, no residual shunt, and no encroachment on the aortic valve. II: A 4-year-old female underwent transcatheter closure of a VSD through the arterial approach. **A** Retrograde left ventriculogram with a 5 F PG catheter in left anterior oblique projection revealed a perimembranous VSD with a right ventricular exit of 0.3 cm. **B** Retrograde crossing with a 5 F cut PG catheter through the VSD over a 0.035-inch/260 Terumo guidewire. **C** Retrograde crossing of a 5 F Lifetech delivery through the VSD over a 0.035-inch/260 guidewire. Then, a retrograde deployment of a 7/5 mm KONAR-MFO device. **D** Control ascending aortogram through a 5 F PG catheter that revealed a well-positioned device, no residual shunt, and no encroachment on the aortic valve

As shown in Table 4, similarly, the VSD patients treated with ADO-I were significantly older and heavier than those receiving KONAR-MFO (*p* < 0.001). The distribution of VSD subtypes differed significantly according to the device used (Figs. 4 and 5). KONAR-MFO was predominantly utilized for perimembranous VSDs (89.3% vs. 44.8%, *p* < 0.001) and was the only device used for Gerbode defects (10.7%, *p* < 0.001). In contrast, muscular and apical VSDs were treated exclusively with ADO-I devices (44.8% and 10.3%, respectively; *p* < 0.001 for both comparisons).

Regarding procedural characteristics, contrast utilization was comparable (*p* = 0.142). However, the KONAR-MFO cohort demonstrated significantly shorter fluoroscopy times (7.4 ± 0.9 vs. 11.8 ± 1.9 min, *p* < 0.001) and procedural durations (41.3 ± 2.7 vs. 47.4 ± 3.4 min, *p* < 0.001), suggesting greater procedural efficiency.

Both devices achieved favorable safety outcomes. Residual shunts, device embolization, and mild tricuspid regurgitation occurred infrequently and did not differ significantly between devices. Complete heart block was observed in 2 (6.9%) ADO-I patients (*p* = 0.026), whereas mild aortic regurgitation occurred exclusively in 3 (10.7%) KONAR-MFO patients (*p* < 0.001). Importantly, no patient required re-intervention, indicating high procedural success and overall safety for both devices when used in appropriately selected VSD anatomies.

**Table 4:** Baseline and procedural characteristics of VSD patients (ADO-I vs. KONAR-MFO)

Variables	ADO-I (*n* = 29)	KONAR-MFO (*n* = 28)	*P*-value
Age (years)	26.4 ± 15.1	3.8 ± 10.5	< 0.001*
Weight (kg)	45.1 ± 11.5	17.9 ± 11.2	< 0.001*
Female gender	23 (97.3%)	24 (85.7%)	0.331
Perimembranous VSD	13 (44.8%)	25 (89.3%)	< 0.001*
Muscular VSD	13 (44.8%)	0 (0.0%)	< 0.001*
Gerbode defect	0 (0.0%)	3 (10.7%)	< 0.001*
Apical VSD	3 (10.3%)	0 (0.0%)	< 0.001*
Fluoroscopy time (min)	11.8 ± 1.9	7.4 ± 0.9	< 0.001*
Procedural time (min)	47.4 ± 3.4	41.3 ± 2.7	< 0.001*
Contrast amount (ml)	56.3 ± 2.2	39.3 ± 4.9	0.142
Residual shunt	1 (3.4%)	1 (3.6%)	0.613
Device embolization	2 (6.9%)	1 (3.6%)	0.427
Complete heart block	2 (6.9%)	0 (0.0%)	0.026*
Mild tricuspid regurgitation	3 (10.3%)	2 (7.1%)	0.237
Mild aortic regurgitation	0 (0.0%)	3 (10.7%)	< 0.001*
Re-intervention	0 (0.0%)	0 (0.0%)	

*ADO-I* Amplatzer duct occluder I, *KONAR-MFO* KONAR-Multifunctional Occluder, *Qp/Qs* Pulmonary-to-systemic flow ratio, *SPAP* Systolic pulmonary artery pressure, *VSD* Ventricular septal defect

P: Significant at ≤ 0.05

## Discussion

As far as we know, this is the first study to compare the use of three types of devices: ADO-I, KONAR-MFO, and ADO-II for transcatheter closure of various phenotypes of PDAs and VSDs. This study demonstrates clear distinctions in patient demographics, clinical presentations, anatomical defect types, and procedural characteristics that influenced device selection and outcomes.

### Demographic, clinical, and hemodynamic findings

Our findings demonstrated that device selection was influenced by the patient’s age and size, with the KONAR-MFO device primarily used in younger and smaller patients, and the ADO-I device favored in older and heavier individuals. This distribution aligns with prior reports indicating that ADO-I, due to its delivery system size and deployment mechanism, is more suitable for larger defects and older and heavier patients [3, 11]. Similarly, some reported cohorts on the KONAR-MFO and ADO-II devices, which are characterized by softer profiles and lower-profile delivery systems, have been increasingly preferred for use in a fairly young population [4, 7, 9, 10]. 

Symptomatology aligned with these demographic patterns; the ADO-I cohort exhibited a higher prevalence of infections and dyspnea, likely reflecting delayed diagnosis and greater hemodynamic burden from larger defects [11], while the KONAR-MFO and ADO-II cohorts were predominantly asymptomatic due to earlier echocardiographic screening in infants and young children [12]. 

Hemodynamically, the ADO-I cohort demonstrated the highest mean Qp/Qs ratio, indicative of more substantial left-to-right shunting; however, systolic pulmonary artery pressures did not differ significantly, suggesting preserved pulmonary vascular adaptation when closure is prior to irreversible pathological pulmonary changes developing [13]. 

### Procedural access and efficiency

Our findings suggest that device selection strongly influences the choice of vascular access, with devices like KONAR-MFO and ADO-II offering greater flexibility and enabling less invasive procedural strategies compared to the ADO-I device. This reflects clear differences in anatomical suitability and possibly operator preferences based on device design characteristics.

The ADO-I device maintained broad applicability across moderate-sized different shapes of PDAs; type A (conical), type C (tubular), type D (saccular), and type E (elongated) along with peri-membranous and muscular VSDs, but was associated with longer procedural and fluoroscopy times, higher contrast use, and more frequent combined arterial and venous accesses, which likely contributed to a higher complication burden, such as hematomas and vascular complications. This is in agreement with the previous reports that used the ADO-I device in variable defects [11]. In contrast, the KONAR-MFO device was predominantly selected for PDA type B (window), peri-membranous VSDs, Gerbode defects, and VSDs with inlet extension, reflecting its importance as a soft double-disc device that promotes wedging and occluding such defects retrogradly. This is consistent with some studies that evaluated the retrograde approach closure of peri-membranous VSDs using the KONAR-MFO devices [9, 14–16]. The flexible structure of the KONAR-MFO device and the availability of its implantation by the retrograde approach allow for modifications during deployment, enabling the operator to move the device away from the aortic valve, minimizing the risk of aortic valve interference, especially in larger defects (> 6 mm) and those with smaller subaortic rims (< 3 mm) [9, 14–16]. However, the ADO-II device was preferentially used in patients with type C (tubular) and type E (elongated) PDA morphologies, indicating a strong device preference for certain PDA morphologies. This is complemented by some studies that highlighted the ADO-II’s advantage in maneuverability due to its flexible left-side disc and suitability for smaller defects [4, 17]. We did not use the ADO-II in any of our VSD patients, as the ADO-II has a more uniform distribution in sizes, and the inlet of the VSD (The left ventricular side) was mostly larger than the exit (the right ventricular side) in all patients, necessitating the use of either the ADO-I or the KONAR-MFO devices.

Procedural efficiency markedly favored the KONAR-MFO and the ADO-II devices, demonstrating shorter procedural and fluoroscopy times with lower contrast use, primarily attributed to their predominant double-disc design with single-access, retrograde deployment strategy without arteriovenous loop formation. The KONAR-MFO offered additional flexibility, facilitating precise device positioning tailored to younger patients and complex defect anatomies, thereby optimizing clinical outcomes. These findings corroborate earlier comparative studies associating ADO-II with shorter procedures and lower radiation exposure [4, 18], as well as recent reports confirming the retrograde KONAR-MFO approach shortens the procedural and fluoroscopy times and offers a safe alternative in cases where creating an arteriovenous loop is challenging or poses additional risks [14–16]. These results support the growing clinical adoption of the KONAR-MFO device as a reliable alternative to surgical repair in small young patients [10]. 

### Success rate and procedural complications

Device deployment was highly successful across the cohort, with re-intervention required in only 5/173 (2.9%) patients, all due to device embolization, which was effectively managed via snare retrieval and re-implantation of a larger device. Regarding complication rates, the KONAR-MFO demonstrated the highest freedom from complications (73.9%), followed by the ADO-I (71.1%) and the ADO-II (66.7%). The ADO-I displayed the highest rate of access site hematoma, likely due to the more frequent requirement of combined arterial and venous accesses, and was additionally associated with mild aortic coarctation, residual shunt, device embolization, and CHB.

The KONAR-MFO showed a low overall complication rate, particularly concerning vascular access-related issues, aligning with the more frequent use via single-only access, mild aortic regurgitation, left LPA stenosis, and mild aortic coarctation, without any of our patients with the KONAR-MFO suffering CHB. This was similar to a retrospective analysis conducted on 70 PDA children in 14 pediatric cardiology centers in 5 countries from 2018 to 2025, with 96.1% safety and efficacy [17], and similar to a nonrandomized, retrospective, multicentre study comprising 333 perimembranous VSD children between 2018 and 2023 [8]. Another study demonstrated that it is a highly effective and versatile device for transcatheter closure of both perimembranous and muscular VSDs across a wide age range, with a low incidence of major complications [8]. Other studies showed favourable mid-term outcomes using KONAR-MFO without significant permanent CHB [9]. However, Bichali et al. reported the first case of delayed CHB with the need for pacemaker implantation 20 months post-procedure with the KONAR-MFO device in a patient with perimembranous VSD [19]. 

The ADO-II presented with femoral artery spasm, residual shunt, and device embolization, possibly reflecting its use in challenging elongated or tubular PDAs. Similar issues have been reported in previous series, particularly when device selection was not strictly guided by defect anatomy, with the majority of complications being minor and resolved conservatively, indicating safety across all devices [13]. 

### PDA subgroup analysis

In this study, the strong association between device type and PDA anatomy is consistent with the current clinical practice, demonstrating that successful PDA closure depends largely on appropriate matching of device design to ductal morphology [7, 11]. 

ADO-II demonstrated the shortest fluoroscopy and procedural times, while ADO-I required the greatest contrast volume and longest procedure duration. This may be attributed to the simpler deployment technique and smaller delivery systems associated with ADO-II. The intermediate procedural metrics observed with KONAR-MFO may reflect the more complex defect anatomy for this device. Similar reductions in fluoroscopy exposure and procedural duration with low-profile devices have been previously described [7, 11]. 

Device embolization occurred only with the ADO-II, and LPA stenosis was observed only with the KONAR-MFO. Although statistically significant, the number of affected patients was very small, and all cases were mild without requiring intervention. Overall, the PDA subgroup analysis confirms that ADO-I, ADO-II, and KONAR-MFO are all effective and safe devices for transcatheter PDA closure when selected according to ductal anatomy.

### VSD subgroup analysis

In this study, the distribution of VSD subtypes differed markedly as KONAR-MFO was predominantly used for perimembranous VSDs and was the only device used for Gerbode defects, whereas muscular and apical VSDs were treated exclusively with ADO-I. These findings are consistent with the structural characteristics of the devices. The symmetrical double-disc design and softer nitinol mesh of the KONAR-MFO facilitate its use in perimembranous defects where preservation of adjacent valvular structures and the conduction system is particularly important [8, 15, 20–22]. In addition, several investigators have reported favorable outcomes using the KONAR-MFO for Gerbode defects owing to its versatility and ability to be delivered through either antegrade or retrograde approaches [23, 24]. 

Although contrast utilization was comparable, fluoroscopy exposure was reduced by approximately 37%, and overall procedural duration was significantly shorter with KONAR-MFO. These findings may be explained by the device’s flexible delivery options, lower-profile system, and the possibility of avoiding complex arteriovenous loop formation in selected cases. Similar reductions in complexity and exposure have been reported in previous studies [8, 15, 20–22]. 

ADO-I and KONAR-MFO devices provided excellent efficacy with very low rates of residual shunting and device embolization. Notably, complete heart block occurred only with the ADO-I; although the number of events was small, this is clinically relevant because conduction disturbances remain a concerning complication of perimembranous VSD closure. Previous studies have suggested that the softer structure and lower radial force of the KONAR-MFO may reduce compression of the atrioventricular conduction tissue and potentially lower the risk of heart block [20, 21]. Conversely, mild aortic regurgitation occurred exclusively with the KONAR-MFO and may be related to the predominance of perimembranous VSDs in this cohort with the proximity of the device to the aortic valve. Importantly, all cases were mild, clinically insignificant, and did not require device removal. Overall, the present findings suggest that both ADO-I and KONAR-MFO are effective and safe for transcatheter VSD closure when selected based on defect morphology and patient characteristics.

### Clinical implications of anatomy-driven device selection

Individualized anatomical clarification, device choice, balancing suitability, and patient characteristics remain fundamental determinants of successful transcatheter intervention and are mandatory to optimize clinical outcomes. Because device allocation was based on operator preference and anatomical suitability rather than randomization, these findings should not be interpreted as evidence of the superiority of one device over another. Rather, they emphasize the importance of individualized device selection to achieve optimal procedural outcomes.

## Conclusions

In this comparative analysis, not only the transcatheter closure devices, but also the patient age, anatomical defect type, clinical presentation, and operator preference influenced device selection and procedural strategies. The ADO-I device maintained broad utility across PDA and muscular VSD closures, but was associated with longer procedural times and increased vascular complications. KONAR-MFO offered versatile applications across varied complex VSD morphologies with favorable safety and procedural profiles, especially in younger, smaller patients. The ADO-II device was preferred for tubular and elongated PDAs, demonstrating efficient procedural metrics. These findings underscore the importance of individualized device choice, balancing anatomical suitability and patient characteristics to optimize outcomes.

## Limitations

This study’s retrospective design inherently limits causal inference. The relatively small sample size of the ADO-II population restricts the generalizability of findings related to this device. Also, the absence of long-term follow-up data precludes assessment of sustained device efficacy and late complications. Prospective, multicenter studies with standardized protocols are needed.

## Data Availability

Our data cannot be shared openly, to protect study participant privacy, the data are availble upon request from the corresponding author.

## Abbreviations

ADO-I: Amplatzer duct occluder I
ADO-II: Amplatzer duct occluder II
ASA: Acetylsalicylic acid
CHB: Complete heart block
CHD: Congenital heart disease
KONAR-MFO: KONAR-multifunctional occluder
LPA: Left pulmonary artery
PDA: Patent ductus arteriosus
Qp/Qs ratio: Pulmonary-to-systemic flow ratio
VSD: Ventricular septal defect
SOB: Shortness of breath
SPAP: Systolic pulmonary artery pressure

## References

[1] Feltes TF, Bacha E, Beekman RH, Cheatham JP, Feinstein JA, Gomes AS et al (2011) Indications for cardiac catheterization and intervention in pediatric cardiac disease: A scientific statement from the AHA. Circulation 123(22):2607–2652. 10.1161/CIR.0b013e31821b1f10 21536996 10.1161/CIR.0b013e31821b1f10

[2] Carminati M, Butera G, Chessa M, Giovanni JD, Fisher G, Gewillig M et al (2007) Transcatheter closure of congenital ventricular septal defects: results of the European Registry. Eur Heart J 28(19):2361–2368. 10.1093/eurheartj/ehm314 17684082 10.1093/eurheartj/ehm314

[3] Pass RH, Hijazi ZM, Hsu DT, Lewis V, Hellenbrand WE (2004) Multicenter USA Amplatzer Patent Ductus Arteriosus Occlusion Device trial. J Am Coll Cardiol 44(3):513–519. 10.1016/j.jacc.2004.04.034 15358013 10.1016/j.jacc.2004.03.074

[4] Yildiz K, Narin N, Oksuz S, Ozdemir R, Pamukcu O, Baykan A et al (2023) Safety and efficacy of Amplatzer duct occluder II and Konar-MF™ VSD occluder in the closure of perimembranous ventricular septal defects in children weighing less than 10 kg. Front Cardiovasc Med 10:1255808. 10.3389/fcvm.2023.1255808 38094116 10.3389/fcvm.2023.1255808 PMC10716692

[5] Odemis E, Kizilkaya MH (2023) Early and mid-term outcomes of transcatheter closure of perimembranous ventricular septal defects using the Lifetech™ Konar-MF Occluder device (MFO) Cardiology in the Young 2023;33: 2021–2026. 10.1017/S1047951122003547 10.1017/S1047951122003547 36380499

[6] Yildiz K, Narin N, Ozdemir R, Oksuz S, Demircan T, Bagli S et al (2023) Closure of transcatheter ventricular septal defect using LifetechTM Konar-MF occluder in children weighing less than 10 kilograms: mid-term results, a tertiary single center experience. Eur Rev Med Pharmacol Sci 27(9):4053–4059. 10.26355/eurrev_202305_32311 37203829 10.26355/eurrev_202305_32311

[7] Lwin N, Ovaert C, Malekzadeh-Milani S, Padovani P, Krasemann Th, Georgiev S et al (2025) Off-label use of the multifunctional occluder for transcatheter patent ductus arteriosus closure: an international experience. J Am Heart Assoc 21;14(20):e043468. 10.1161/JAHA.125.043468 10.1161/JAHA.125.043468 PMC12684515 41120817

[8] Haddad RN, Houeijeh A, Odemis E, Thambo JB, Saritas T, Ece I et al (2025) MIOS-MFO, a multicenter international observational study of the Lifetech KONAR-MF ventricular septal defect occluder in treating perimembranous ventricular septal defects. Rev Esp Cardiol (Engl Ed) 78(11):947–956. 10.1016/j.rec.2025.02.010 40058523 10.1016/j.rec.2025.02.010

[9] Elafifi A, Kotit S, Shehata M, Deyaa O, Ramadan A, Tawfik M (2025) Early experience with transcatheter ventricular septal defects closure with the KONAR-MF multifunctional occluder. Front Pediatr. 10.3389/fped.2025.1528490. 7;131528490 40260313 10.3389/fped.2025.1528490 PMC12009802

[10] Sinha R, Shrivastava S, Aggarwal N (2017) Clinical outcomes of patent ductus arteriosus closure in adults: Long-term follow-up. Heart Asia 9(2):e010947. 10.1136/heartasia-2017-010947 29470559 10.1136/heartasia-2017-010947 PMC5818050

[11] Faella HJ, Hijazi ZM (2000) Closure of the patent ductus arteriosus in adults with the Amplatzer duct occluder: Immediate and long-term results. Catheter Cardiovasc Interv 50(4):508–514. 10.1002/1522-726X(200011)50:4<508::AID-CCD20>3.0.CO;2-S 10.1002/1522-726x(200009)51:1<50::aid-ccd11>3.0.co;2-6 10973018

[12] Fortescue EB, Lock JE, Galvin T, McElhinney DB (2010) Trends in outcomes of patent ductus arteriosus closure in infants and children. Circ Cardiovasc Interv 3(2):152–159. 10.1111/j.1747-0803.2010.00435.x

[13] Haddad RN, Saliba ZS (2023) Comparative outcomes of two competitive devices for retrograde closure of perimembranous ventricular septal defects. Front Cardiovasc Med 5:10:1215397. 10.3389/fcvm.2023.1215397 10.3389/fcvm.2023.1215397 PMC10354815 37476569

[14] Vuran G, Yılmazer MM, Murat M, Karahan C, Bilen M, Karaçelik M et al (2025) Optimized retrograde approach and device selection with Konar-MF™ for pediatric transcatheter ventricular septal defect closure. Turkish J Thorac Cardiovasc Surg 33(3):301–311. 10.5606/tgkdc.dergisi.2025.27178 10.5606/tgkdc.dergisi.2025.27178 PMC12421569 40936991

[15] Kuswiyanto RB, Gunawijaya E, Djer MM, Noormanto, Rahman MA, Murni IK et al (2022) Transcatheter Closure of Perimembranous Ventricular Septal Defect Using the Lifetech Konar-Multi Functional Occluder: Early to Midterm Results of the Indonesian Multicenter Study. Global Heart 17(1):15. 10.5334/gh.1106 35342698 10.5334/gh.1106 PMC8877696

[16] Laha S, Gangopadhyay D, Roy M, Singh A, Nandi D, Dutta J (2024) Procedural outcomes of percutaneous closure of perimembranous and other ventricular septal defects using Konar-MF occluder and short-term follow-up. Ann Pediatr Cardiol 17(2):101–108. 10.4103/apc.apc_201_23 39184123 10.4103/apc.apc_201_23 PMC11343394

[17] Jiang D, Zhang J, Fan Y, Han B, Zhao L, Yi Y et al (2021) The efficacy and medium to long-term follow-up of transcatheter retrograde closure of perimembranous ventricular septal defects via the femoral artery with Amplatzer duct occluder II in children. Front Pediatr 9:571407. 10.3389/fped.2021.571407 34113582 10.3389/fped.2021.571407 PMC8185017

[18] Ghaderian M, Ramezani S, Behdad S, Gharipour M, Dianatkhah M, Hovesepian S et al (2024) Safety and efficacy of using amplatzer ductal occluder type I and II for peri membranous ventricular septal defect closure: A systematic review. Arya Atherosclerosis J 20(6):54–64. 10.48305/arya.2024.42641.2962 10.48305/arya.2024.42641.2962 PMC11913456 40103627

[19] Bichali S, Houeijeh A, Godart (2024) Late complete atrioventricular block after transcatheter perimembranous ventricular septal defect closure with the KONAR-MF™ VSD occluder: a case report and systematic review. Cardiol Young 34(11):2480–2483. 10.1017/S1047951124026891 39473223 10.1017/S1047951124026891

[20] Kabadayı B, Bengi Eren Z, Odemis E (2025) Efficacy and safety of the LifeTech™ Multifunctional Occluder (Konar-MFO) in transcatheter closure of ventricular septal defects: a systematic review and meta-analysis. Catheter Cardiovasc Interve 2025;106(1):325–335. 10.1002/ccd.31560 10.1002/ccd.31560 40275637

[21] Tanidir IC, Baspinar O, Saygi M, Kervancioglu M, Guzeltas A, Odemis E (2020) Use of Lifetech™ Konar-MF, a device for both perimembranous and muscular ventricular septal defects: A multicentre study. Int J Cardiol 310:43–50. 10.1016/j.ijcard.2020.02.056 32122701 10.1016/j.ijcard.2020.02.056

[22] Laha S, Gangopadhyay D, Roy M, Singh A, Nandi D, Dutta J (2024) Procedural outcomes of percutaneous closure of perimembranous and other ventricular septal defects using Konar-MF occluder and short-term follow-up. Ann Pediatr Cardiol 20(2):101–108. 10.4103/apc.apc_201_23 10.4103/apc.apc_201_23 PMC11343394 39184123

[23] Boukaram L, Haddad RN, Daou L, Saliba Z (2021) Transcatheter closure of congenital Gerbode-type ventricular septal defect using the new multifunctional occluder device. J Card Surg 36(8):2986–2988. 10.1111/jocs.15624 34021626 10.1111/jocs.15624

[24] Haddad RN, Boudjemline Y, Combes N, Hadeed Kh, Karsenty C, Saliba Z (2022) Three centers experience with device closure of congenital Gerbode-type perimembranous ventricular septal defects. J Card Surg 37:2714–2724. 10.1111/jocs.16713 35771212 10.1111/jocs.16713

